# Gene expression of the heat stress response in bovine peripheral white blood cells and milk somatic cells in vivo

**DOI:** 10.1038/s41598-020-75438-2

**Published:** 2020-11-05

**Authors:** J. B. Garner, A. J. Chamberlain, C. Vander Jagt, T. T. T. Nguyen, B. A. Mason, L. C. Marett, B. J. Leury, W. J. Wales, B. J. Hayes

**Affiliations:** 1 Agriculture Victoria Research, Animal Production Sciences, Ellinbank Dairy Centre, Ellinbank, VIC 3821 Australia; 2 Agriculture Victoria Research, AgriBio, Centre for AgriBiosciences, Bundoora, VIC 3083 Australia; 3 DataGene LTD., AgriBio, 5 Ring Road, Bundoora, VIC 3083 Australia; 4 Faculty of Veterinary and Agricultural Sciences, The University of Melbourne, Parkville, VIC 3052 Australia; 5 Queensland Alliance for Agriculture and Food Innovation, Centre for Animal Science, University of Queensland, St Lucia, QLD 4067 Australia; 6 Centre for Agriculture Innovation, School of Agriculture and Food, Faculty of Veterinary and Agricultural Sciences, The University of Melbourne, Parkville, 3052 Australia

**Keywords:** Molecular biology, Physiology, Genetics, Agricultural genetics, Animal breeding, Gene expression, Genomics, Sequencing

## Abstract

Heat stress in dairy cattle leads to reduction in feed intake and milk production as well as the induction of many physiological stress responses. The genes implicated in the response to heat stress in vivo are not well characterised. With the aim of identifying such genes, an experiment was conducted to perform differential gene expression in peripheral white blood cells and milk somatic cells in vivo in 6 Holstein Friesian cows in thermoneutral conditions and in 6 Holstein Friesian cows exposed to a short-term moderate heat challenge. RNA sequences from peripheral white blood cells and milk somatic cells were used to quantify full transcriptome gene expression. Genes commonly differentially expressed (DE) in both the peripheral white blood cells and in milk somatic cells were associated with the cellular stress response, apoptosis, oxidative stress and glucose metabolism. Genes DE in peripheral white blood cells of cows exposed to the heat challenge compared to the thermoneutral control were related to inflammation, lipid metabolism, carbohydrate metabolism and the cardiovascular system. Genes DE in milk somatic cells compared to the thermoneutral control were involved in the response to stress, thermoregulation and vasodilation. These findings provide new insights into the cellular adaptations induced during the response to short term moderate heat stress in dairy cattle and identify potential candidate genes (*BDKRB1* and *SNORA19*) for future research.

## Introduction

Depending on the severity, heat stress can be a major inhibitor of milk production in dairy cows leading to significant negative economic implications for the dairy industry. The mechanisms involved in the decline in milk yield are multifactorial and include systemic and intracellular functions^1,2^. The heat stress induced decline in milk yield can only be partially explained by the reduction in feed intake. Pair feeding experiments have established that the proportion of the milk yield decline directly related to nutrient intake is estimated to be between 30 and 50%, and the remaining proportion attributed to the direct effects of heat stress on post-absorptive metabolism, nutrient partitioning, insulin sensitivity and the cellular pathway responses for cell survival^3,4^. It is yet to be determined if transcriptional changes in mammary epithelial cells are directly involved in the milk yield decline induced by heat stress.

When cells are exposed to heat stress, the cellular stress response function is initiated which limits damage and supports cell recovery^5^. If the stress exceeds the cell’s ability to limit damage, the highly conserved process of apoptosis is initiated to remove damaged cells and maintain tissue function^6^. The heat shock proteins (*HSP*) are a primary component of the cellular stress response and the increased gene expression of these proteins has been observed in response to thermal stress in humans, rats, chickens, and cattle^7–10^. Experiments in vitro have identified changes in the expression of these genes in response to heat stress in peripheral white blood cells and mammary epithelial cells^11,12^. Peripheral blood is an accessible source of transcriptional information relevant to metabolism and the immune system. Bovine peripheral white blood cells respond to heat shock in vitro by reducing DNA synthesis and increasing synthesis of *HSP72*^11^. Peripheral white blood cells from Sahiwal heifers had altered gene expression in peripheral white blood cells after exposure to 42 °C for 4 h. Changes included activation of heat shock transcription factor 1 (*HSF1*), increased expression of *HSP* and decreased expression of immune system activation via extracellular expression of *HSP*^12^. Additionally, mammary epithelial cells exposed to heat shock in vitro upregulated genes associated with the stress response and protein repair and an overall downregulation of genes regulating biosynthesis, metabolism, and morphogenesis in the mammary epithelial cells^1,9,10^. Experiments in vitro provide useful insights into the bovine cellular responses to heat stress, however the in vivo responses are yet to be described for lactating Holsteins.

In order to measure gene expression responses in vivo, non-invasive sampling is important to minimise the impact on the experimental animals. The transcriptome information obtained from both milk somatic cells and from mammary tissue cells have high similarities indicating that milk somatic cells can be used as a suitable alternative to mammary cells in understanding the gene expression responses of the mammary gland^13^. Mammary cells are obtained by a highly invasive biopsy of the mammary gland whereas peripheral blood and milk are easily accessible and minimally invasive sources of somatic cells. The cells present in milk are a heterogenous population of lymphocytes, neutrophils, macrophages and exfoliated epithelial cells^14^. These cells represent important components of the innate immune defence mechanisms of the mammary gland^15^. Peripheral white blood cells isolated from blood are a robust source of systemic transcriptome information. Peripheral white blood cells and milk somatic cells are suitable tissues to assess the systemic (blood) and local (mammary gland) gene expression changes induced by heat stress.

The objectives of this investigation were to determine the gene expression profiles, using RNA sequences in peripheral white blood cells and milk somatic cells, before and during a heat challenge (for the same cows). Then compare these gene expression profiles to control cows exposed to thermoneutral conditions in controlled-climate chambers, and to describe the results in the context of the physiology and production responses during a heat challenge. The hypotheses were (1) that the in vivo gene expression in peripheral white blood cells of heat challenged cows would be different compared to that occurring in the peripheral white blood cells of thermoneutral control cows, and (2) that the in vivo gene expression in milk somatic cells of heat challenged cows would be different compared to that occurring in the milk somatic cells of thermoneutral control cows. As there is limited evidence directly comparing the peripheral white blood cells and milk somatic cells gene expression changes during heat stress we propose the null hypothesis (3) that the in vivo gene expression in peripheral white blood cells and milk somatic cells of heat challenged and thermoneutral control cows will be similar.

## Results

On average, 88 million reads were generated per white blood cell library and 174 million reads per milk cell library. Ninety-two % of reads passed quality control, of which 93% were mapped to the reference genome. One milk sample was excluded from the count matrix as less than 80% of reads mapped to the reference genome, resulting in 12 cows in the peripheral white blood cells dataset, and 11 cows in the milk somatic cells dataset. The total numbers of genes with counts that passed quality control from the peripheral white blood cell and milk somatic cell libraries were 19,020 and 20,040, respectively.

### Differential gene expression

**Figure 1:**
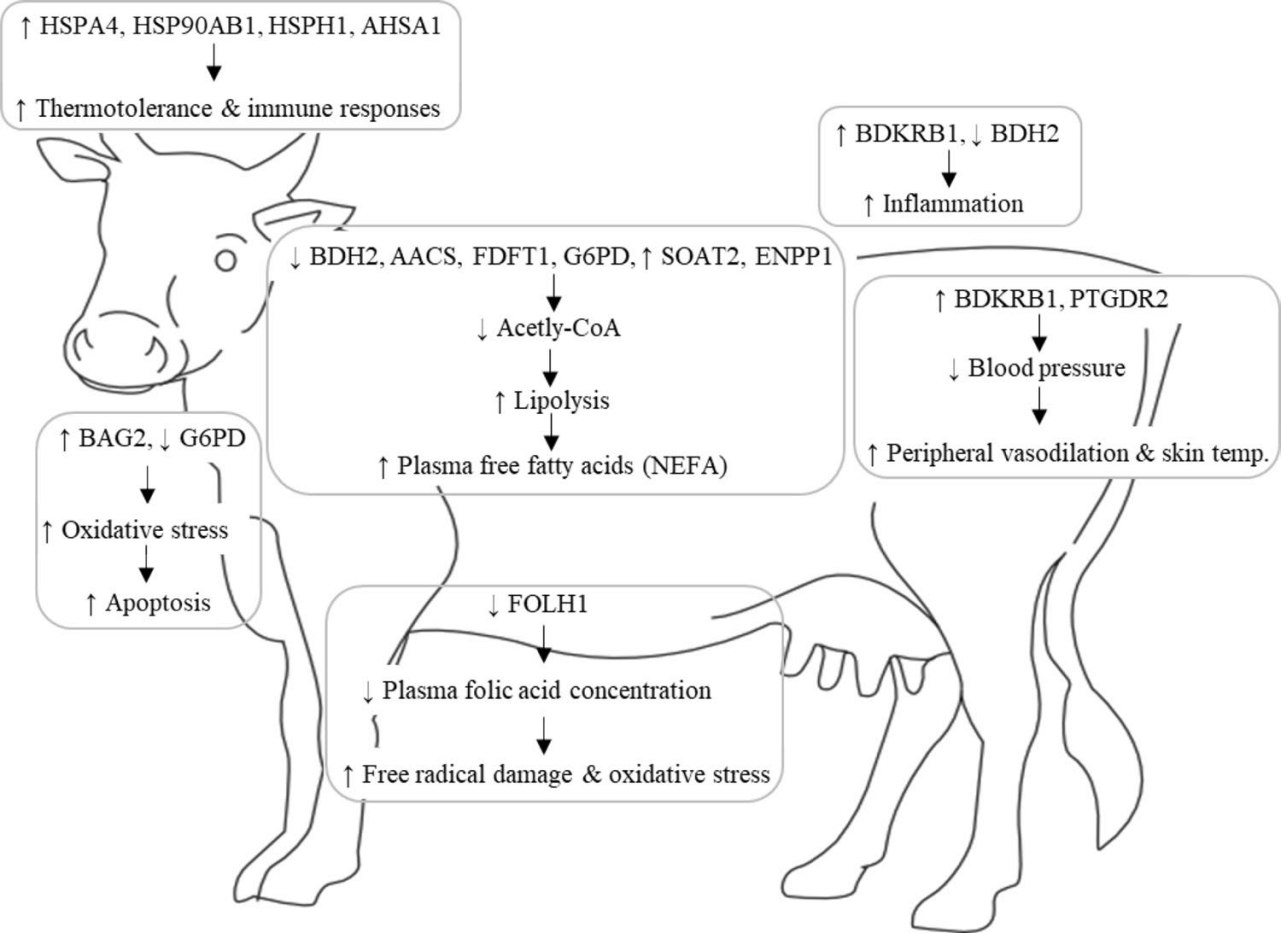
Summary schematic of the genes differentially expressed in response to the heat challenge and the key physiological processes associated with these genes.

**Figure 2:**
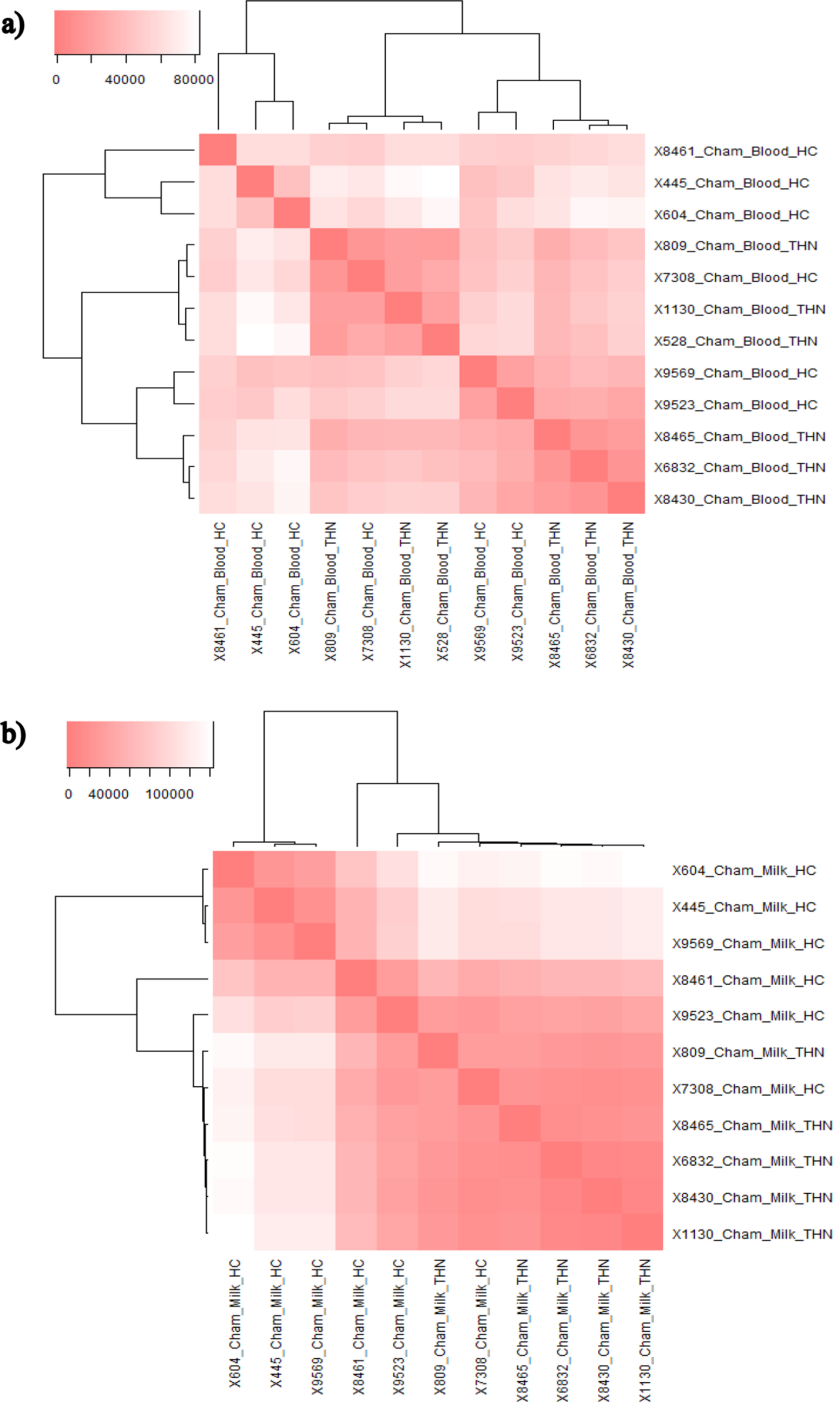
(**a**) Heatmap showing clustering of samples of peripheral white blood cells between the HC and THN control cows, it is noted that there is some overlap between the treatments. (**b**) Heatmap showing clear clustering of samples of milk somatic cells between the HC and THN control cows. Heatmaps were generated using the R packages gplots and heatmap3 (R: A Language and Environment for Statistical Computing, R Foundation for Statistical Computing, Vienna Austria, Version 3.3.2, 2016). Heatmap colour intensity indicates that the more intense the colour the more similar the samples.

There were 926 genes differentially expressed (*P* < 0.05, before correction for multiple testing using nominal *P* values) between the thermoneutral (THN) and heat challenge (HC) treatment groups during the heat challenge in the peripheral white blood cells (499 downregulated and 427 upregulated, Supplementary Table 1), 129 of these genes matched unnamed transcripts. There were 469 genes differentially expressed milk somatic cells (*P* < 0.05, before correction for multiple testing using nominal *P* values) between the THN and HC treatment groups during the heat challenge (286 downregulated and 183 upregulated, Supplementary Table 2), with 85 of these genes matched unnamed transcripts. Figure 1 summarises the differential gene expression across both peripheral white blood cells and milk somatic cells and the key physiological responses during the heat challenge. The heat map plot shows the hierarchical clustering of the THN and HC samples in both the peripheral white blood cells and milk somatic cells (Fig. 2a,b). The clustering of the peripheral white blood cells does indicate some overlap between the treatment samples (Fig. 2a), however the clustering of the milk somatic cells samples shows clear separation of the treatments with only two samples overlapping (Fig. 2b). During the experiment there were no cases of clinical mastitis and there was no difference between the mean somatic cell counts (SCC) of the treatment groups for the duration of the experiment (THN SCC = 217,000 cells/ml and HC SCC = 185,000 cells/ml).

Differentially expressed genes were corrected for multiple testing (*P* < 0.01 and 5% false discovery rate) to identify the most differentially expressed genes in each tissue to focus on the key differences between the THN and HC groups during the heat challenge. The genes that remained significant after multiple testing correction (*P* < 0.01 and 5% false discovery rate) were investigated in detail to determine the relevant pathways associated with heat stress physiology. This list included 9 downregulated and 6 upregulated in peripheral white blood cells (Table 1), and 6 downregulated and 9 upregulated in the milk somatic cells (Table 2). Differentially expressed genes in both tissues were analysed using pathway analysis.

**Table 1:** Differentially expressed genes in peripheral white blood cells during the heat challenge and their expression in terms of fold change compared to the thermoneutral controls.

Biological process	Gene ID	Gene	Description	Fold change	*P*-value
GO:0006813 potassium ion transport	ENSBTAG00000012798	*KCNH8*	Potassium voltage-gated channel, subfamily H member 8	− 5.1293	2.73238E-05
GO:0006635 fatty acid beta-oxidation	ENSBTAG00000002526	*BDH2*	3-hydroxybutyrate dehydrogenase 2	− 1.8470	4.38571E-07
GO:0005215 transporter activity	ENSBTAG00000005031	*DOC2A*	Double C2-like domain alpha	− 1.3069	0.000328931
GO:0007586 digestion	ENSBTAG00000021565	*PRSS2*	Serine protease 2	− 1.1665	9.13122E-05
GO:0006281 DNA repair	ENSBTAG00000004769	*NEIL2*	Nei like DNA glycosylase 2	− 0.9366	0.0004
GO:0055114 oxidation reduction	ENSBTAG00000012432	*FDFT1*	Farnesyl-diphosphate farnesyltransferase 1	− 0.9300	0.0010
GO:0006629 lipid metabolism	ENSBTAG00000005678	*AACS*	Acetoacetyl-CoA synthetase	− 0.8918	0.0002
GO:0008270 zinc ion binding	ENSBTAG00000014172	*FGD2*	FYVE, RhoGEF and PH domain containing 2	− 0.8787	0.0004
GO:0005515 protein binding	ENSBTAG00000019290	*PACSIN2*	Protein kinase C and casein kinase substrate in neurons 2	− 0.4358	0.0006
GO:0003676 nucleic acid binding	ENSBTAG00000004080	*TIAL1*	TIA1 cytotoxic granule-associated RNA binding protein-like 1	0.1293	0.0007
GO:0008152 metabolism	ENSBTAG00000021830	*ENPP1*	Ectonucleotide pyrophosphatase/phosphodiesterase 1	0.2761	0.0005
GO:0005520 insulin-like growth factor binding	ENSBTAG00000008389	*HTRA1*	HtrA serine peptidase 1	0.7468	0.0010
GO:0004089 carbonate dehydratase activity	ENSBTAG00000017969	*CA4*	Carbonic anhydrase 4	1.0000	0.0004
NA*	ENSBTAG00000043816	*SNORA19*	Small nucleolar RNA SNORA19	1.3219	7.47E-11
GO:0032496 response to lipopolysaccharide	ENSBTAG00000007052	*BDKRB1*	Bradykinin receptor B1	5.0000	0.0001

*Gene is not matched to a known biological process.

**Table 2:** Differentially expressed genes in milk somatic cells during the heat challenge and their expression in terms of fold change compared to the thermoneutral controls.

Biological process	Gene ID	Gene	Description	Fold change	*P*-value
GO:0003677 DNA binding	ENSBTAG00000014741	*OTX2*	Orthodenticle homeobox 2	− 1.8480	0.0014
GO:0008083 growth factor activity	ENSBTAG00000012413	*FGF12*	Fibroblast growth factor 12	− 1.2861	0.0030
GO:0003824 catalytic activity	ENSBTAG00000021899	*PDE9A*	Phosphodiesterase 9A	− 1.1787	0.0009
GO:0004866 endopeptidase inhibitor activity	ENSBTAG00000011975	*SERPINB1*	Serpin family B member 1	− 0.8950	0.0002
GO:0008610 lipid biosynthesis	ENSBTAG00000007798	*DGAT2L6*	Diacylglycerol O-acyltransferase 2-like 6	− 0.7225	0.0026
GO:0003676 nucleic acid binding	ENSBTAG00000012086	*CNOT8*	CCR4-NOT transcription complex, subunit 8	− 0.5700	0.0014
GO:0019722 calcium-mediated signalling	ENSBTAG00000039652	*PTGDR2*	prostaglandin D2 receptor 2	0.4767	0.0006
GO:0034619 cellular chaperone-mediated protein complex assembly	ENSBTAG00000015683	*HSPA4*	heat shock protein family A (HSP70) member 4	0.5076	0.0011
GO:0043065 positive regulation of apoptosis	ENSBTAG00000012586	*HSPD1*	Heat shock protein family D (Hsp60) member 1	0.5365	0.0006
GO:0006950 response to stress	ENSBTAG00000020477	*AHSA1*	activator of HSP90 ATPase activity 1	0.8683	0.0014
GO:0008270 zinc ion binding	ENSBTAG00000013615	*CHORDC1*	Cysteine and histidine-rich domain containing 1	0.8690	0.0000
GO:0006950 response to stress	ENSBTAG00000006025	*AHSA2*	Activator of HSP90 ATPase homolog 2	1.0462	0.0006
GO:0005515 protein binding	ENSBTAG00000015692	*HSPA4L*	Heat shock protein family A (Hsp70) member 4 like	1.8772	0.0033
GO:0015485 lipid metabolism	ENSBTAG00000005623	*SOAT2*	Sterol O-acyltransferase 2	1.6667	0.0062
GO:0005524 ATP binding	ENSBTAG00000025442	*HSPA1L*	Heat shock 70 kDa protein 1-like	2.0862	0.0002

### Differentially expressed genes in peripheral white blood cells

The biological processes associated with downregulated genes in peripheral white blood cells in response to the heat challenge were fatty acid beta-oxidation, oxidation reduction, potassium ion transport and digestion, and biological processes associated with upregulated genes were related to response to lipopolysaccharide, nucleic acid binding and insulin-like growth factor signalling (Tables 1 and 3). The differentially expressed genes in peripheral white blood cells were significantly related to eleven biological pathways including ketone body synthesis and degradation, calcium signalling, butanoate metabolism (Supplementary Table 3).

**Table 3:** Biological process associated with differentially expressed genes in peripheral white blood cells between the heat challenge and thermoneutral treatment groups.

Biological process	*P*-value	q-value*
GO:0006508 proteolysis	2.67E-04	0.001287
GO:0008610 lipid biosynthesis	0.0064	0.01
GO:0043089 positive regulation of Cdc42 GTPase activity	1.10E−06	1.67E−05
GO:0030574 collagen catabolism	1.15E−04	6.76E−04
GO:0006635 fatty acid beta-oxidation	3.00E−04	0.001325
GO:0006284 base-excision repair	6.05E−04	0.001945
GO:0007586 digestion	0.0014	0.0034
GO:0006289 nucleotide-excision repair	0.0015	0.0034
GO:0009239 enterobactin biosynthesis	0.0016	0.0034
GO:0035023 regulation of Rho protein signal transduction	0.0069	0.0105
GO:0043087 regulation of GTPase activity	0.01	0.0137
GO:0007156 homophilic cell adhesion	0.0105	0.0139
GO:0006730 one-carbon compound metabolism	0.0117	0.0145
GO:0006813 potassium ion transport	0.0432	0.038
GO:0006281 DNA repair	0.0437	0.038
GO:0045806 negative regulation of endocytosis	0.0063	0.01
GO:0042733 embryonic digit morphogenesis	0.0085	0.0124
GO:0017145 stem cell division	0.0095	0.0134
GO:0043537 negative regulation of blood vessel endothelial cell migration	0.0095	0.0134
GO:0030514 negative regulation of BMP signalling pathway	0.0137	0.0163
GO:0030512 negative regulation of transforming growth factor beta receptor signalling pathway	0.0158	0.0174
GO:0001937 negative regulation of endothelial cell proliferation	0.0179	0.0192
GO:0016525 negative regulation of angiogenesis	0.021	0.0218
GO:0006695 cholesterol biosynthesis	0.0283	0.027
GO:0016126 sterol biosynthesis	0.0324	0.0301
GO:0008299 isoprenoid biosynthesis	0.0365	0.0333
GO:0032496 response to lipopolysaccharide	0.0375	0.0337
GO:0006885 regulation of pH	0.0487	0.0403

*q-value is the *P*-value corrected for multiple testing using Molecule Annotation System, MAS 3.

### Differentially expressed genes in milk somatic cells

Biological processes associated with downregulated genes in milk somatic cells in response to the heat challenge were DNA binding, growth factor activity, lipid biosynthesis, and endopeptidase inhibition, and biological processes associated with upregulated genes were the stress response, apoptosis, and lipid metabolism (Tables 2 and 4). The differentially expressed genes in milk somatic cells were significantly related with four biological pathways which were affected by the heat challenge including antigen processing and presentation, Wnt signalling and Type I diabetes (Supplementary Table 4).

**Table 4:** Biological process associated with differentially expressed genes in milk somatic cells between the heat challenge and thermoneutral treatment groups.

Biological process	*P*-value	q-value*
GO:0006950 response to stress	0.0088	0.0068
GO:0002368 B cell cytokine production	2.87E−13	3.36E−12
GO:0002842 positive regulation of T cell mediated immune response to tumour cell	2.87E−13	3.36E−12
GO:0042026 protein refolding	1.43E−12	1.17E−11
GO:0043032 positive regulation of macrophage activation	4.29E−12	2.71E−11
GO:0032733 positive regulation of interleukin-10 production	1.00E−11	5.13E−11
GO:0032735 positive regulation of interleukin-12 production	6.00E−11	2.34E−10
GO:0002755 MyD88-dependent toll-like receptor signalling pathway	1.41E−10	4.45E−10
GO:0050821 protein stabilization	2.04E−10	5.97E−10
GO:0048291 isotype switching to IgG isotypes	3.89E−10	9.59E−10
GO:0032755 positive regulation of interleukin-6 production	3.89E−10	9.59E−10
GO:0042100 B cell proliferation	7.74E−09	1.59E−08
GO:0006986 response to unfolded protein	2.08E−08	4.05E−08
GO:0006919 caspase activation	3.14E−08	5.48E−08
GO:0043065 positive regulation of apoptosis	3.24E−05	4.28E−05
GO:0043066 negative regulation of apoptosis	3.45E−05	4.29E−05
GO:0032727 positive regulation of interferon-alpha production	4.09E−10	9.59E−10
GO:0032729 positive regulation of interferon-gamma production	1.48E−07	2.34E−07
GO:0006367 transcription initiation from RNA polymerase II promoter	4.95E−06	7.51E−06
GO:0050870 positive regulation of T cell activation	3.35E−05	4.29E−05
GO:0042110T cell activation	2.30E−04	2.62E−04
GO:0006935 chemotaxis	0.0069	0.0055
GO:0034619 cellular chaperone-mediated protein complex assembly	0.0015	0.0014
GO:0045745 positive regulation of G-protein coupled receptor protein signalling pathway	0.0023	0.002
GO:0070096 mitochondrial outer membrane translocase complex assembly	0.0023	0.002
GO:0007193 G-protein signalling adenylate cyclase inhibiting pathway	0.0061	0.0051
GO:0019722 calcium-mediated signalling	0.0234	0.0163

*q-value is the *P*-value corrected for multiple testing using Molecule Annotation System, MAS 3.

### Common genes differentially expressed in peripheral white blood cells and milk somatic cells

A venn diagram analysis^16^ of the data on a global scale (before correction for multiple testing), identified 50 genes in peripheral white blood cells and milk somatic cells that are commonly differentially expressed during a thermoneutral period compared to a heat challenge (Supplementary Fig. 1). Out of these 50 genes, 42 matched annotated sequences and 8 matched unnamed or hypothetical transcripts. In the peripheral white blood cells, 17 genes were downregulated and 33 were upregulated, and in the milk somatic cells 23 genes were downregulated and 27 were upregulated (Supplementary Table 5). Gene ontology analysis was used to identify the commonly differentially expressed pathways of the 42 genes common to both tissues.

The most upregulated gene in both cell types was the heat shock protein family A (*HSP70*) member 6 (*HSPA6*), with a 2.1 fold increase in peripheral white blood cells (P = 0.013) and 2.3 fold increase in milk somatic cells (P = 0.008, Supplementary Table 5). The most downregulated gene in both cell types was folate hydrolase 1 (*FOLH1B*) with a − 2.7 fold decrease in peripheral white blood cells (P = 0.01) and − 2.74 fold decrease in milk somatic cells (P = 0.04, Supplementary Table 5). Genes downregulated in peripheral white blood cells and milk somatic cells in response to the heat challenge in both tissues were associated with carbohydrate metabolism, oxidative stress and proteolysis. Genes upregulated in both tissues were related to apoptosis, oxidation reduction, and the stress response (Supplementary Table 5).

## Discussion

This experiment is the first in vivo investigation into the gene expression responses of lactating Holstein–Friesian cows to a short-term moderate heat challenge in controlled-climate chambers. The cows exposed to the heat challenge in this experiment experienced hyperthermia, with significant increases in rectal and vaginal temperatures, skin surface temperatures, respiration rate and panting score, and a decline in milk production and feed intake compared to the thermoneutral controls^17^. A whole transcriptome analysis was conducted to describe gene expression changes of lactating cows in response to a heat challenge in controlled-climate chambers in peripheral white blood cells and milk somatic cells. This discussion focuses on the most differentially expressed genes in each tissue and the genes commonly expressed in both peripheral white blood cells and milk somatic cells to describe changes in relevant biological processes and pathways in the context of heat stress physiology.

There were significant differentially expressed genes between the heat challenge and the thermoneutral control groups in the peripheral white blood cells, indicating that the heat challenge induced changes in gene expression supporting our first hypothesis. The gene expression of peripheral white blood cells describes some complex changes in the expression of genes related to major shifts in the metabolism of the heat stressed cow. Bradykinin receptor B1 (*BDKRB1*) was the most highly upregulated gene in peripheral white blood cells. The increased expression of this gene infers that the rate of inflammation was accelerated by the heat challenge. *BDKRB1* is transiently induced by tissue injury and inflammation, such as pro-inflammatory cytokine release, immune cell flux, and increased vascular permeability^18^. The expression of 3-hydroxybutyrate dehydrogenase 2 (*BDH2*) also provides supporting evidence that inflammation was a key biological response affected by the heat challenge. *BDH2* was the second most downregulated gene expressed in peripheral white blood cells by the heat stressed cows. *BDH2* has been shown to be downregulated in human macrophage cells in response to inflammation and endoplasmic reticulum stress in vitro^18^. Changes in the expression of *BDH2* are generally implicated with modulating iron-limiting innate immune responses^19^. The altered regulation of *BDKRB1* and *BDH2* indicate that inflammation was a key biological pathway affected by the heat challenge in peripheral white blood cells.

The altered gene expression of lipid metabolism related genes, in conjunction with the plasma metabolite analysis^17^, suggest that fatty acid mobilisation occurred at a rate greater than it could be metabolised in the cows exposed to the heat challenge. Under thermoneutral conditions, a cow experiencing negative energy balance would have an increase in plasma non-esterified fatty acids (NEFA) and the ketone, beta-hydroxy butyrate (BHB), due to a reduction in available glucose. Ketone bodies are produced in the liver once fatty acids have been converted to acetyl-CoA via the processes of beta oxidation^20^. Key functions of the genes *BDH2* and acetoacetyl-CoA synthetase (*AACS*), are fatty acid beta-oxidation and the synthesis and degradation of ketone bodies. The downregulation of *BDH2* and *AACS* in the cows exposed to the heat challenge is related to the significant increase in plasma NEFA concentrations in response to the heat challenge^17^. Thus, during the heat challenge, the lipid metabolism responses were not consistent with negative energy balance driven by insufficient energy intake as there was an absence of elevated plasma BHB concentration, despite the increase in plasma NEFA concentrations. It can be speculated that acetyl-CoA was limiting in the Krebs cycle, resulting in fatty acids not being oxidised by the liver and converted to ketones at the same rate that adipose tissue was being mobilised by lipolysis. To confirm these findings, a further investigation into post-absorptive metabolism of dairy cows exposed to a short-term moderate heat challenge would be necessary.

The heat challenge affected the expression of genes involved in lipid and carbohydrate metabolism in addition to genes involved in fatty acid beta-oxidation. Farnesyl-diphosphate farnesyltransferase 1 (*FDFT1*), a key regulator of lipid metabolism and cholesterol biosynthesis, was downregulated in the peripheral white blood cells of heat stressed cows. *FDFT1* encodes squalene synthase that catalyses the production of squalene from farnesyl pyrophosphate and is the first step in the sterol biosynthesis pathway^21^. Furthermore, the expression of ectonucleotide pyrophosphatase/phosphodiesterase 1 (*ENPP1*) was increased during heat stress and this enzyme has been shown to influence insulin sensitivity and resistance and obesity in humans^22^. Transgenic mice over-expressing *ENPP1* had hyperglycaemia and hyperinsulinemia and reduced glucose uptake in the muscle^22^. *ENPP1* has been suggested to play a role in pathophysiological changes associated with insulin resistance such as hyperglycaemia through elevated hepatic gluconeogenesis^23^. The expression of these genes indicates that fundamental changes in lipid and carbohydrate metabolism occurred during heat stress which are not consistent with the normal metabolism changes that occur during periods of negative energy balance under thermoneutral conditions.

Heat stress induces changes in the cardiovascular system, and the expression of genes in the peripheral white blood cells are related to cardiovascular physiology responses measured during the heat challenge. Bradykinin receptor B1 (*BDKRB1*) is involved in the regulation of blood pressure. Our findings of increased expression of *BDKRB1* in heat stressed dairy cows suggests that the heat challenge may have induced hypotension in these cows*.* Experimentally induced hypotension results in an increase in the expression of *BDKRB1* as it assumes a hemodynamic role^24^. During heat stress, the demand for elevated flow of blood to the skin (peripheral vasodilation) is increased to allow for heat dissipation through the skin which causes a sudden drop in blood pressure, or hypotension^25^. Indeed, *Bos taurus* cattle exposed to 40 °C had higher cardiac output and lower blood pressure than at 15 °C due to increased peripheral vasodilation^26^. Skin surface temperature is linked to the changes in skin blood flow and is a function of peripheral vasoconstriction (cooler skin temperature)^27^ and peripheral vasodilation (hotter skin temperature)^28^. The surface temperature of the skin of cows exposed to the heat challenge was higher than that of the control cows under thermoneutral conditions^17^, indicative of an increase in peripheral vasodilation and heat dissipation through the skin. As the upregulation of the *BDKRB1* in peripheral white blood cells is linked to the heat induced reduction in blood pressure and an increase in peripheral vasodilation, we propose that *BDKRB1* has a potential role as a novel biological indicator of heat stress tolerance in dairy cows.

A potential novel biomarker of rectal temperature heat stress responses was identified in this experiment. Small nucleolar RNA SNORA19 (*SNORA19)* is an unannotated gene which has been associated with stabilising cellular function and RNA metabolism during stress as well a major quantitative trait locus (QTL) related to variation in rectal temperature in cattle^29^. The expression of this gene was increased in peripheral white blood cells in response to the heat challenge, supporting this genes potential as a candidate for predicting rectal temperature in dairy cattle and a reason why this gene warrants further investigation.

There were significant differentially expressed genes in the milk somatic cells of cows in the thermoneutral control group and the heat challenged cows, indicating that the heat challenge induced changes in gene expression supporting our second hypothesis. The cows exposed to the heat challenge experienced a 53% reduction in milk yield and a decline in milk quality from their baseline production levels^17^. The gene expression in milk somatic cells collected from heat stressed cows highlight some similar themes to those found in peripheral white blood cells. However, a notable difference between milk somatic cells and peripheral white blood cells was the expression of heat shock proteins. Cellular chaperones and heat shock proteins including *HSP60 (HSPD1), HSP 70* genes *(HSPA4, HSPA4L and HSPA1L),* and activators of *HSP90 (AHSA1 and AHSA2)*, were all upregulated in the milk somatic cells during the heat challenge. These genes are well known to be crucial in the cellular stress response to heat and have a role in protein folding, protein degradation and cell survival^30^. This highlights the differences in the local (mammary gland) and systemic (blood) responses to heat stress and the susceptibility of the mammary gland to cellular stress during heat exposure. We speculate that changes in the gene expression of heat shock proteins in milk somatic cells might directly regulate the cascade of physiological responses that leads to heat stress induced decline in milk production.

The development of thermotolerance is a key physiological process to increase cell survival to heat exposure. The expression of thermotolerance following heat exposure is considered to occur within several hours and can persist for 3–5 days^31^. This is supported by an in vitro experiment investigating bovine mammary cells exposed to heat shock which increased the expression of molecular chaperone genes and returned to baseline expression levels 8 h after heat exposure^1^. In the current experiment, the increased expression of these heat shock protein genes by day four of the heat challenge indicates that this effect which was previously observed in vitro, does indeed occur in vivo. Consequently, our observations provide in vivo evidence that cells in the mammary gland adapt physiological processes to achieve a level of thermotolerance.

*HSP70* is a key gene in the development of thermotolerance in the mammary gland, and its gene expression is induced by heat shock, oxidative stress, ischemia and inflammation^32^. *HSP70* was upregulated in response to heat stress in buffalo mammary epithelial cells in vitro^10^. The regulation of *HSP70* production is crucial for cell survival and represents the generalised molecular process induced by almost every cell in an organism and the response of this gene to heat stress can differ between individuals and this is a factor in their ability to develop thermotolerance^32^. The changes in the expression of significant genes regulating the development of thermotolerance signify the susceptibility of the mammary gland cells to the deleterious effects of heat exposure. The expression of HSP’s is also linked to changes in the immune system as they are important activators of the innate immune system through the function of cytokines^33^. *HSP70* genes induce antibody production and T cell activation^34^. The *HSP70* genes were upregulated in response to the heat challenge, therefore antibody production was potentially increased to improve immune functions under stress. Thus, in this experiment, in response to the heat challenge, the mammary gland developed thermotolerance and increased immune responses mediated by the action of *HSP70*.

In the milk somatic cells, the heat challenge induced the expression of a gene which regulates vasodilation and involved in thermoregulation. The pharmacological activities of prostaglandin D2 receptor 2 (*PTGDR2*) include vasodilation and bronchoconstriction^35–37^. Other functional roles of *PTGDR2* are in thermoregulation and fever response^38^. The increased expression of *PTGDR2* in milk somatic cells could be related to the level of peripheral vasodilation that occurs in the mammary gland as a thermoregulation mechanism. Peripheral vasodilation is a key response to reduce body temperature by utilising the temperature gradient from the skin to the ambient air. The udder surface temperature measured during the heat challenge was higher than the surface temperature measured on the neck and flank and was significantly higher than the corresponding surface temperatures measured on the thermoneutral control cows^17^. The heat challenge induced changes in vasodilation of the mammary gland as evidenced by the increased expression of *PTGR2* in the milk somatic cells and skin surface temperatures, is presumably an adaptive response to maintain thermoregulation of the mammary gland.

There were common genes significantly differentially expressed in both cell types during the heat challenge compared to the corresponding gene expressions measured in the thermoneutral control animals, and accordingly, we reject the third hypothesis. To determine the biological pathways affected by the heat challenge. the common genes differentially expressed in both the peripheral white blood cells and milk somatic cells were investigated. The genes differentially expressed in both tissues which had a fold change in the same direction are discussed. The heat shock protein transcripts *HSPA4*, *HSPA6, HSPA1A, HSPA1L (HSP70), HSP90AB1 (HSP90), HSPH1 (HSP 105)* and *AHSA1*, *AHSA2* (activator of *HSP90*) were all upregulated in response to the heat challenge in both the peripheral white blood cells and milk somatic cells. *HSPH1* was the most highly upregulated gene in both the peripheral white blood cells and milk somatic cells during the heat challenge. The expression of *HSP’s* is a well-documented cellular response to thermal stress. Our in vivo findings are supported by other experiments which measured an upregulation of *HSP105* in response to heat stress in bovine peripheral white blood cells^12^ and buffalo mammary epithelial cells in vitro^10^. Additionally, in bovine mammary epithelial cells *HSP70* was upregulated in response to heat stress in vitro^9^*,* and a threefold increase in *HSP70* in blood lymphocytes from *Bos taurus* heifers was measured in response to heat stress^39^. The increase in the expression of the *HSP* genes provides evidence of the responsiveness of peripheral white blood cells and milk somatic cells to heat stress in vivo and indicates that the cellular stress response was occurring at the systemic level and locally in the mammary gland.

Apoptosis has been implicated to be a critical physiological response to thermal stress at the cellular level. BCL2- associated athanogene 2 *(BAG2)* has pro-apoptotic properties and possesses a nucleotide exchange mechanism with *HSP70*, which forms a *BAG-HSP70* complex that coordinates cellular processes including stress signalling, cell division, cell death and differentiation^40^. In our in vivo experiment*, BAG2* was upregulated in response to heat stress in both tissues which illustrates that apoptosis was a key biological process initiated during heat stress in peripheral white blood cells and milk somatic cells. The reduced expression of glucose 6-phosphate dehydrogenase *(G6PD)* is also related to an increase in oxidative stress and the rate of apoptosis. Reactive oxygen species generated in oxidative metabolism cause damage to cells, leading to cell death^41^. Cells with reduced expression of *G6PD* have low antioxidant defence and are especially sensitive to oxidative stress due to low steady state levels of glutathione^42^. The oxidative stress response is also mediated by the expression of stress induced phosphoprotein 1 (*STIP1*). The primary function of this response is to activate the expression of antioxidant genes. *STIP1,* also known as HSP70/HSP90 organising protein, plays a crucial antioxidant role under stress conditions. This gene has been proposed as a biomarker for heat stress in dairy cows due to its increased expression activity in adipose tissue during summer^43^. The expression of this gene in the present experiment was upregulated in both peripheral white blood cells and milk somatic cells, supporting *STIP1* as a potential biomarker for heat stress in dairy cows. The altered expression patterns of apoptosis and oxidative stress related genes indicates that the heat challenge increased the vulnerability of peripheral white blood cells and milk somatic cells to free radical damage from oxidative stress.

Antioxidants are free radical scavengers which protect the bodies defence system against the accumulation of free radicals produced during heat stress^44^. Folate hydrolase 1B (*FOLH1B*) acts as an important physiological antioxidant^45^, hydrolyses dietary folate and modulates plasma folate status^46^. *FOLH1* also facilitates methylation to form methionine, the biosynthesis of amino acids and deoxynucleotides required for DNA repair and replication^47^. Plasma folic acid concentration can decrease during heat stress and has been used as a dietary supplement to mitigate heat stress in quails^48^, and to reduce oxidative stress of mouse embryos during heat stress^45^. *FOLH1* was the most highly downregulated gene in both peripheral white blood cells and milk somatic cells during the heat challenge, we can speculate that in our experiment, during the heat challenge, there may have been a reduction in plasma folic acid concentration, but this would need to be confirmed with further research. A reduction in plasma folic acid can increase the susceptibility of cells to oxidative stress due to reduced antioxidant activity of folic acid. Oxidative stress is characterised by various deleterious processes caused by imbalances between excessive reactive oxygen species or reduced antioxidant defences^48^. High ambient temperature can increase the production of oxygen derived free radicals. To combat this, antioxidants such as vitamin C have been fed to dairy cows during heat stress and have resulted in improvements in heat stress tolerance^49^. The significant downregulation of *FOLH1* during heat stress in both peripheral white blood cells and milk somatic cells suggests that folic acid, offered as a dietary supplement, could reduce the oxidative stress caused during heat stress in dairy cattle.

Major shifts in glucose metabolism are well established responses of lactating dairy cows to thermal stress^4^. Glucose 6-phosphate dehydrogenase *(G6PD*) is involved in metabolism of glucose and carbohydrates and plays an important role in ruminants’ lipogenesis as it provides the essential compounds of NADPH for the synthesis of fatty acids catalysing the first reaction in the pentose phosphate pathway^50^. During the heat challenge this gene was downregulated in both peripheral white blood cells and milk somatic cells, compared to baseline expression levels. *G6PD* is utilised in glycolysis to produce energy in the form of adenosine triphosphate and nicotinamide adenine dinucleotide, which are stored in the form of glycogen, or used in the pentose phosphate pathway. *G6PD* is generally activated after a decrease in the amount of nicotinamide adenine dinucleotide phosphate, and other positive regulators of this gene include vitamin D, insulin and S6 kinase^50^. The downregulation of *G6PD* in the heat stressed cows suggests that glucose was not limiting. This is supported by the plasma concentrations of glucose measured during the heat challenge which were not different between heat stressed and thermoneutral exposed cows^17^. Furthermore, the reduced expression of *G6PD* is linked with hyperglycaemia and diabetes^51^. We can speculate that glucose availability was not limiting biochemical processes, and that other factors were influencing the characteristic metabolic disruptions during heat stress.

In conclusion, the differential gene expression analysis of peripheral white blood cells and milk somatic cells of cows exposed to a controlled heat challenge compared to thermoneutral conditions provides detailed insights into the cellular adaptations induced during a heat stress response. This experiment identified that a four-day heat challenge in controlled-climate chambers altered the expression of many heat responsive genes in peripheral white blood cells and milk somatic cells and that many of these genes are involved in major biological processes. The findings of this experiment have enabled the linking of transcriptional changes, i.e. gene expression to changes in physiological responses. Comprehensive changes in gene expression determined by RNA sequencing, highlight the responsiveness of peripheral white blood cells and milk somatic cells to environmental stress. Furthermore, this study has shown that the sampling of white blood cells and milk somatic cells provides a non-invasive in vivo model to understand the cellular adaptations induced systemically and in the mammary gland of dairy cattle during thermal stress. The important novel aspect of this experiment was that it identified for the first time in vivo, potential candidate genes for heat stress including *BDKRB1* and *SNORA19*, fundamental changes to lipid metabolism, the cardiovascular system and the role of heat shock proteins in cellular protection of the mammary gland during heat stress.

## Materials and methods

### Animals and experimental design

The experiment received animal ethics approval from the Agricultural Research and Extension Animal Ethics Committee of the Department of Economic Development, Jobs, Transport and Resources, Victoria, Australia. All experimental procedures were performed in accordance with the animal ethics approval and regulations. Detailed experimental design, methods and measurements are previously reported^17^. In brief, twelve multiparous non-pregnant Holstein Friesian cows (mean ± SD; 6.4 ± 1.02 years of age, 3.8 ± 1.07 lactations, 261 ± 24.9 days in milk; 638 ± 34.7 kg live weight) were randomly assigned to thermoneutral control (THN) or heat challenge (HC) treatment in two groups of six cows each containing 3 THN, and 3 HC exposed cows. Within each group, cows were then randomly assigned to 1 of 6 controlled-climate chambers. The experiment consisted of 2 periods including, (1) 7-day baseline period in ambient conditions; (2) 4-day treatment period of THN or HC in controlled-climate chambers. The experiment was conducted in late autumn to negate any potential influence of physiological acclimation, during the baseline period the cows experienced ambient weather conditions ranging from daily average temperature of 8.7 °C (range of 4.1–13.2 °C) and average relative humidity of 89.1% (range of 66.7–99% RH), and average THI of 52.2 (range of 47–58), at the Ellinbank Dairy Centre, Victoria, Australia.

### Controlled-climate chambers

The conditions in the controlled-climate chambers for the HC treatment were designed to impose a moderate level of heat stress, the temperature-humidity index (THI) remained above 74 and did not exceed 84 (details reported by Garner et al. 2017). The cows in the HC treatment experienced daily cyclical temperatures and relative humidity, ranging from 21.3 to 32.8 °C and 35 to 88% RH (THI 69–83) for the 4-day duration. The bovine thermoneutral range was maintained for the THN treatment (10.2–15.9 °C, and 61–94% relative humidity, THI 55).

### Sampling and measurements

Blood samples were taken by coccygeal venepuncture at 1600 h into two vacutainers containing EDTA, once on day 5 of the baseline period, and once on day 4 of the treatment period, immediately after the afternoon milking and feeding. Blood samples were placed immediately on ice and centrifuged at 531 g for 15 min at 4 °C, then processed according to the blood fractionation and white blood cell stabilisation procedure in the RiboPure blood kit (Ambion by Life Technologies) protocol, and stored in RNAlater (Invitrogen by Thermo Fisher Scientific, Life Technologies) at -20 °C.

Milk samples were collected 1 h following the afternoon milking, on day 5 of the baseline period, and once on day 4 of the treatment period. Milk was collected from all four quarters of the udder. Two 50 mL aliquots of each milk sample were transported to the laboratory on ice. Somatic cells were isolated from milk following a similar method to that reported by Boutinaud et al.^52^, with the following modifications. 50 µL of 0.5 M EDTA was added to each milk aliquot and then centrifuged at 531 g at 4 °C for 10 min. Cream and skim milk were discarded, leaving the cell pellet. The cell pellet was washed with 10 mL PBS solution with 0.5 mM EDTA and again centrifuged for 10 min at 4 °C. The cell pellets from duplicate tubes were resuspended and combined. The entire suspension was then transferred to a 2 mL microcentrifuge tube, spun down, the supernatant was pipetted off and then the cell pellet was resuspended in 200 µL of PBS solution, and 1 mL of RNAlater (Invitrogen by Thermo Fisher Scientific, Life Technologies) and stored at − 20 °C.

Cows were milked twice daily at 0600 and 1500 h, with yields recorded automatically in the milking parlour at each milking during the baseline period (MM25; DeLaval International, Tumba, Sweden). Whilst in the controlled-climate chambers, the cows were milked using an inbuilt milking system (same clusters and pulsators as the milking parlour) and milk yields were recorded manually, details are presented in Garner et al.^17^.

### RNA extraction and sequencing

RNA was extracted from peripheral white blood cells using RiboPure Blood Kit (Ambion) according to manufacturer’s instructions. RNA was extracted from milk somatic cells using Trizol Plus RNA Purification Kit (Ambion) according to manufacturer’s instructions. RNA quality was assessed using the BioAnalyser 2100 (Agilent Technologies, Palo Alto, CA) and concentrations were determined using a NanoDrop ND-1000 spectrophotometer (NanoDrop Technologies, Rockland, DE).

RNAseq libraries were prepared using the SureSelect Strand Specific RNA Library Prep Kit (Agilent) according to manufacturer’s instructions. Each library was uniquely barcoded, randomly assigned to one of four pools and sequenced on a HiSeq 3000 (Illumina Inc) in a 150-cycle paired-end run. One hundred fifty base paired-end reads were called with bcltofastq and output in fastq format. Sequence quality was assessed using FastQC. QualityTrim (https://bitbucket.org/arobinson/qualitytrim) was used to trim and filter poor quality bases and sequence reads. Adaptor sequences and bases with quality scores less than 20 were trimmed from the end of reads. Reads with mean quality scores less than 20, or greater than 3 N, or greater than three consecutive bases with a quality scores less than 15, or final length less than 50 bases were discarded. Only paired reads were retained for alignment.

### Sequence alignment and count matrix generation

Paired RNA reads that passed quality checks were aligned to the Ensembl UMD3.1 bovine genome assembly using TopHat2^53^ allowing for two mismatches. Alignment files (.bam) for white blood cell and milk cell libraries with > 12.5 and 25 million read pairs, respectively (after quality control filtering) with > 80% mapping rate were retained for gene count matrix generation. Gene counts for the alignment files were created using the python package HTSeq^54^. Counts were combined to form a gene by sample count matrix. This count matrix was then normalised to consider library size using the R software package DESeq^55^ (R: A Language and Environment for Statistical Computing, R Foundation for Statistical Computing, Vienna Austria, Version 3.3.2, 2016).

### Data analysis

To determine the source of differential expression, the following linear mixed model was fitted for each gene:

$$\mathrm{y}=\mathbf{X}\mathrm{b}+\mathbf{Z}\mathrm{u}+\mathrm{e}$$

where y = ln(1 + read count) is the vector of the log transformed normalised numbers of RNASeq reads that were mapped to genes; b is the vector of fixed effects (including library pool: pool 1 and pool 2, group: group 1 and group 2, treatment: thermoneutral (THN) and heat challenge (HC), parity: 5 levels, age of cow (in years) as a covariate, sampling period (baseline or treatment), interaction between treatment and sampling period, days in milk as a covariate, and milk yield on the day of sampling as covariate; u is the vector of cow ID as a random effect (fitted to capture the cow effect with the repeated measurement of gene expression before and during the heat challenge); **X** and **Z** are design matrices for fixed and random effects, respectively; e is the residual. The variance components were estimated using ASReml^56^. Differential expression between the THN and HC groups was investigated using the size of this effect, and *P* values for this effect corrected for multiple testing. *P* values were determined significant using the Benjamini–Hochberg procedure^57^ to correct for multiple testing. A false discovery rate of 5% was applied to nominal *P* values. All nominal *P* values with a Benjamini–Hochberg adjusted *P* value less than the 0.05 false discovery rate were deemed as significant. All *P* values presented are nominal and have been corrected for multiple testing with the false discovery rate of 5%. Fold change was calculated using the average expression of each gene between the THN and HC groups and is expressed as log_2_. A negative fold change indicates a downregulation of the gene and a positive fold change indicates an upregulation of the gene.

The normalised mapped read counts were also used to assess overall similarity between samples, by generating heat maps. This was undertaken using the R software (R: A Language and Environment for Statistical Computing, R Foundation for Statistical Computing, Vienna Austria, Version 3.3.2, 2016,) packages: hclust and heatmap.

### Gene identification and biological pathway analysis

Data was analysed using a Molecule Annotation System (MAS 3) https://bioinfo.capitalbio.com/mas3//, to determine the biological pathways and gene ontology terms (GO) associated with the differentially expressed genes. MAS 3 maps the input gene list into matching entities in various biological databases including KEGG, Genbank, Gene Ontology, BioCarta, GenMapp, UniGene and describes pathway analysis and provides confidence *q* values^12^. Gene annotation was conducted using Ensembl https://asia.ensembl.org/Bos_taurus/Info/Index.

## Supplementary information

Supplementary Tables.

Supplementary Figure.

## Data Availability

All RNAseq data and sample metadata are available from NCBI Sequence Read Archive using BioProject accession PRJNA616134.
