# Supplementary material for: Gene expression of the heat stress response in bovine peripheral white blood cells and milk somatic cells in vivo
Source: Sci Rep. 2020 Nov 5;10:19181. doi: 10.1038/s41598-020-75438-2 (PMC7645416; doi:10.1038/s41598-020-75438-2)
Supplement: Supplementary file 2 — Supplementary Figure. [file 41598_2020_75438_MOESM2_ESM.pdf]

**Gene expression of the heat stress response in bovine peripheral white blood cells and milk somatic cells *in vivo***

Garner, J. B.[1,*], Chamberlain, A.J. [2], Van der Jagt, C. [2], Nguyen, T. T. T. [2], Mason, B.A. [2], Marett, L. C.[1,5], Leury, B. J.[3], Wales, W. J.[1,5], Hayes, B. J [4, 2].

[1] Agriculture Victoria, Animal Production Sciences, Ellinbank Dairy Centre, Ellinbank, Victoria 3821, Australia

[2] Agriculture Victoria, AgriBio, Centre for AgriBiosciences, Bundoora, Victoria 3083, Australia

[3] Faculty of Veterinary and Agricultural Sciences, The University of Melbourne, Parkville 3052, Victoria, Australia.

[4] Queensland Alliance for Agriculture and Food Innovation, Centre for Animal Science, University of Queensland, St Lucia, Queensland 4067, Australia.

[5] Centre for Agriculture Innovation, School of Agriculture and Food, Faculty of Veterinary and Agricultural Science, The University of Melbourne, Parkville 3010, Australia

[*] Corresponding author: josie.garner@agriculture.vic.gov.au

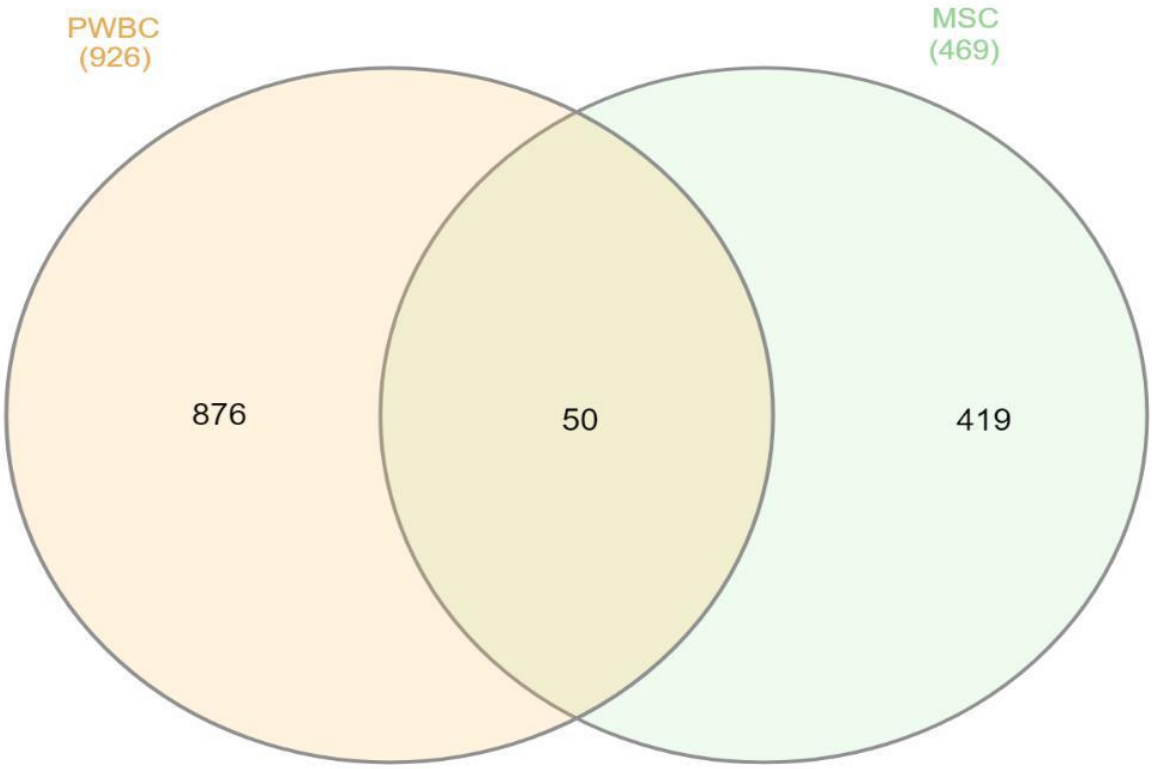


**Supplementary Figure 1.** Venn diagram showing the number of common differentially expressed genes between PWBC and MSC during the heat challenge.
